# 2,3-Dihydroxybenzoate *meta*-Cleavage Pathway is Involved in *o*-Phthalate Utilization in *Pseudomonas* sp. strain PTH10

**DOI:** 10.1038/s41598-018-38077-2

**Published:** 2019-02-04

**Authors:** Daisuke Kasai, Takumi Iwasaki, Kazuki Nagai, Naoto Araki, Tatsunari Nishi, Masao Fukuda

**Affiliations:** 1 Department of Bioengineering, Nagaoka University of Technology, Nagaoka, Niigata 940-2188 Japan; 2 Genaris, Inc., Yokohama, Kanagawa 230-0046 Japan; 3 Present Address: Department of Biological Chemistry, Chubu University, Kasugai, Aichi 487-8501 Japan

## Abstract

*Pseudomonas* sp. strain PTH10 can utilize *o*-phthalate which is a key intermediate in the bacterial degradation of some polycyclic aromatic hydrocarbons. In this strain, *o*-phthalate is degraded to 2,3-dihydroxybenzoate and further metabolized via the 2,3-dihydroxybenzoate *meta*-cleavage pathway. Here, the *opa* genes which are involved in the *o*-phthalate catabolism were identified. Based on the enzymatic activity of the *opa* gene products, *opaAaAbAcAd*, *opaB*, *opaC*, and *opaD* were found to code for *o*-phthalate 2,3-dioxygenase, dihydrodiol dehydrogenase, 2,3-dihydroxybenzoate 3,4-dioxygenase, and 3-carboxy-2-hydroxymuconate-6-semialdehyde decarboxylase, respectively. Collectively, these enzymes are thought to catalyze the conversion of *o*-phthalate to 2-hydroxymuconate-6-semialdehyde. Deletion mutants of the above *opa* genes indicated that their products were required for the utilization of *o*-phthalate. Transcriptional analysis showed that the *opa* genes were organized in the same transcriptional unit. Quantitative analysis of *opaAa*, *opaB*, *opaC*, *opaD*, *opaE*, and *opaN* revealed that, except for *opaB* and *opaC*, all other genes were transcriptionally induced during growth on *o*-phthalate. The constitutive expression of *opaB* and *opaC*, and the transcriptional induction of *opaD* located downstream of *opaB*, suggest several possible internal promoters are existence in the *opa* cluster. Together, these results strongly suggest that the *opa* genes are involved in a novel *o*-phthalate catabolic pathway in strain PTH10.

## Introduction

Phthalate isomers including *o*-phthalate (OPA), terephthalate, and isophthalate have been widely used as plasticizers to impart favorable properties such as flexibility and softness to various polymers. Furthermore, they are key intermediates in the bacterial degradation of phthalate esters, as well as some polycyclic aromatic hydrocarbons found in fossil fuels. They represent hazardous endocrine-disrupting substances capable of binding to the estrogen receptor^1–3^, which makes their release into the environment a serious concern worldwide^4^. Microbial degradation represents a promising technology for solving environmental contamination by phthalate isomers. OPA is known as a major intermediate metabolite and a key substance in the microbial degradation of phthalate esters^5,6^ and some polycyclic aromatic hydrocarbons, such as fluoranthene^7^, fluorene^8^, and phenanthrene^9^. The OPA catabolic pathway has been reported in many gram-negative and gram-positive bacteria. In gram-negative bacteria, including *Burkholderia cepacia* DBO1^10^, *Pseudomonas fluorescens* PHK^11^, and *Comamonas testosteroni*^12^, OPA is reportedly oxidized by OPA 4,5-dioxygenase to form *cis*-4,5-dihydroxy-4,5-dihydrophthalate; the latter is then converted to 4,5-dihydroxyphthalate by a dehydrogenase. The resulting product is further transformed to protocatechuate by a decarboxylase, before entering the aromatic ring-cleavage pathway. In gram-positive bacteria such as *Mycobacterium vanbaalenii* PYR-1^13^, *Arthrobacter keyseri* 12B^14^, *Terrabacter* sp. strain DBF63^15^, and *Rhodococcus jostii* RHA1^16^, OPA is oxidized by OPA 3,4-dioxygenase to yield *cis*-3,4-dihydroxy-3,4-dihydrophthalate, which is then converted to 3,4-dihydroxyphthalate by a dehydrogenase. The resulting product is again transformed to protocatechuate by a decarboxylase and further catabolized through the aromatic ring-cleavage pathway.

Microbial degradation of terephthalate and isophthalate has been reported in several bacteria^5,17–19^. *C. testosteroni* YZW-D metabolizes terephthalate and isophthalate through catabolic pathways that involve the *tph* and *iph* genes, respectively^20^. These catabolic genes have been also characterized in *Comamonas* sp. strain E6, which is seemingly able to degrade terephthalate and isophthalate^17,21^. In these catabolic pathways, terephthalate and isophthalate are converted to protocatechuate and further degraded via the protocatechuate 4,5-cleavage pathway.

Of the catabolic pathways involved in the degradation of phthalate isomers, only the one proceeding via protocatechuate has been identified so far. In the present study, an OPA-assimilating bacterium, *Pseudomonas* sp. strain PTH10, was isolated. This strain is able to degrade OPA via 2,3-dihydroxybenzoate (23DHBA). However, not all the genes responsible for OPA degradation via 23DHBA have been characterized. This study is the report describing the identification of genes involved in the conversion of OPA to 23DHBA and implying the OPA degradation pathway via 23DHBA in strain PTH10. These results will improve our understanding of the OPA utilization system in the environmental microorganisms.

## Results

### Isolation and characterization of an OPA-degrading strain

An OPA-degrading mixed culture was enriched from soil that had been treated with 1 mM OPA. Strain PTH10 isolated from this enrichment culture was able to grow on OPA as the sole carbon and energy source; however, it was unable to utilize terephthalate and isophthalate. The 16S rRNA gene (1,537 bp) sequence which was found by the draft genome sequence analysis of strain PTH10 showed similarity with those of the *Pseudomonas* genus.

High-performance liquid chromatography (HPLC) analysis revealed the production of 23DHBA during growth of strain PTH10 on OPA. When the cells grown on OPA were incubated with 23DHBA, the reaction mixture turned yellow, suggesting the release of a *meta*-cleavage compound (data not shown). These results indicate that in this strain, OPA is degraded through the 23DHBA *meta*-cleavage pathway. However, no growth of this strain on 23DHBA was found. It seemed to be caused by lack of a positive uptake of 23DHBA in this strain.

### Identification of 23DHBA *meta*-cleavage pathway genes

To identify the OPA degradation genes in strain PTH10, we first attempted to isolate the 23DHBA 3,4-dioxygenase gene. The 23DHBA *meta*-cleavage pathway in *Pseudomonas reinekei* MT1 has been reported by Marín and coworkers^22^. The extradiol ring cleavage dioxygenase, 23DHBA 3,4-dioxygenase (DhbA; accession number, AFN52421), is responsible for ring cleavage of 23DHBA in this species. To find the corresponding gene in strain PTH10, the draft genome sequence of this strain was determined and a tBLASTn homology search using the amino acid sequence of DhbA as the query. A gene, named *opaC*, that encoded a DhbA homolog was found. The amino acid sequence of OpaC showed 55% and 26% identity with those of DhbA and 2,3-dihydroxy-*p*-cumate 3,4-dioxygenase of *Pseudomonas putida* KL47 (CmtC; accession number, ABA10796), respectively (Table 1). Based on sequence similarity, the *opaC* gene product is thought to be involved in the extradiol ring cleavage of 23DHBA.

**Table 1:** Characteristics of *opa* genes.

Gene	Deduced molecular mass (Da)^a^	Representative homolog	Identity (%)^b^	Accession no.
*opaAa*	48,985	Anthranilate 1,2-dioxygenase large subunit (AntA) from *Acinetobacter baylyi* ADP1	30	AAC34813
	(433)	Benzoate 1,2-dioxygenase large subunit (BenA) from *A. baylyi* ADP1	30	AAC46436
*opaAb*	19,403	Anthranilate 1,2-dioxygenase small subunit (AntB) from *A*. *baylyi* ADP1	27	AAC34814
	(171)	Benzoate 1,2-dioxygenase small subunit (BenB) from *A. baylyi* ADP1	26	AAC46437
*opaAc*	11,911	Carbazole 1,9a-dioxygenase ferredoxin component (CarAc) from *Pseudomonas resinovorans* CA10	47	BAA21733
	(110)	Anthranilate 1,2-dioxygenase ferredoxin component (AndAb) from *Burkholderia cepacia* DBO1	36	AAO83641
*opaAd*	36,575	Acenaphthene dioxygenase ferredoxin reductase component (ArhA4) from *Sphingomonas* sp. strain A4	46	BAE93942
	(337)	Carbazole 1,9a-dioxygenase ferredoxin reductase component (CarAd) from *P. resinovorans* CA10	42	BAA21735
*opaB*	29,067	Benzoate diol dehydrogenase (BenD) from *A*. *baylyi* ADP1	50	AAC46439
	(269)	1,2-Dihydroxycyclohexa-3,4-diene carboxylate dehydrogenase (XylL) from *Pseudomonas putida* mt-2 (pWW0)	49	AAA26050
*opaC*	32,431	DBA 3,4-dioxygenase (DhbA) from *Pseudomonas reinekei* MT1	55	AFN52421
	(285)	2,3-Dihydroxy-*p*-cumate 3,4-dioxygenase (PsbC2) from *Rhodopseudomonas palustris* no. 7	51	BAA82122
*opaD*	36,819	2-Amino-3-carboxymuconate 6-semialdehyde decarboxylase (NbaD) from *Pseudomonas fluorescens* KU-7	30	BAC65312
	(327)	5-Carboxy-2-hydroxymuconate-6-semialdehyde decarboxylase (PraH) from *Paenibacillus* sp. strain JJ-1b	28	BAH79106
*opaE*	52,750	HMS dehydrogenase (NahI) from *P. putida* G7 (NAH7)	75	BAE92168
	(486)	HMS dehydrogenase (XylG) from *P. putida* mt-2 (pWW0)	71	AAA26053
*opaF*	7,356	OCA tautomerase (DhbF) from *P. reinekei* MT1	44	AFN52426
	(66)	OCA tautomerase (DmpI) from *Pseudomonas* sp. strain CF600	38	CAA43229
*opaG*	28,313	OCA decarboxylase (DmpH) from *Pseudomonas* sp. strain CF600	89	CAA43228
	(264)	OCA decarboxylase (XylI) from *P. putida* mt-2 (pWW0)	87	AAA25693
*opaH*	30,679	HMS hydrolase (DmpD) from *Pseudomonas* sp. strain CF600	66	CAA36993
	(276)	HMS hydrolase (DhbI) from *P. reinekei* MT1	59	AFN52439
*opaI*	27,888	HPD hydratase (XylJ) from *P. putida* mt-2 (pWW0)	71	AAA26055
	(261)	HPD hydratase (DhbD) from *P. reinekei* MT1	63	AFN52424
*opaJ*	37,358	HOV aldolase (DhbH) from *P. reinekei* MT1	89	AFN52428
	(345)	HOV aldolase (XylK) from *P. putida* mt-2 (pWW0)	87	AAA25692
*opaK*	32,998	Acetaldehyde dehydrogenase (DhbG) from *P. reinekei* MT1	76	AFN52427
	(312)	Acetaldehyde dehydrogenase from (NbaJ) from *P. fluorescens* KU-7	68	BAC65307
*opaL*	37,594	TRAP transporter solute receptor from *Virgibacillus halodenitrificans*	29	CDQ31851
	(349)	Putative periplasmic binding protein from *Aromatoleum aromaticum*	27	CAI10584
*opaM*	44,696	Phosphate-selective porin from *Alcanivorax pacificus* W11-5	61	EKE36862
	(412)	Phosphate-selective porin from *Methylophaga thiooxydans* DMS010	33	EEF79304
*opaN*	70,229	TRAP transporter from *Roseobacter litoralis* Och 149	38	AEI95128
	(660)	TRAP transporter from *Bacillus wakoensis* JCM 9140	33	GAE27593
*opaO*	32,346	Hypothetical protein from *Halobacterium* sp. strain DL1	29	EHB57276
	(300)	Hypothetical protein from *Natrialba magadii* ATCC 43099	28	ADD06240

^a^The values in parentheses are the numbers of amino acid residues.

^b^Percentages of identity by aligning the deduced amino acid sequences by use of EMBOSS alignment tool.

**Figure 1:**
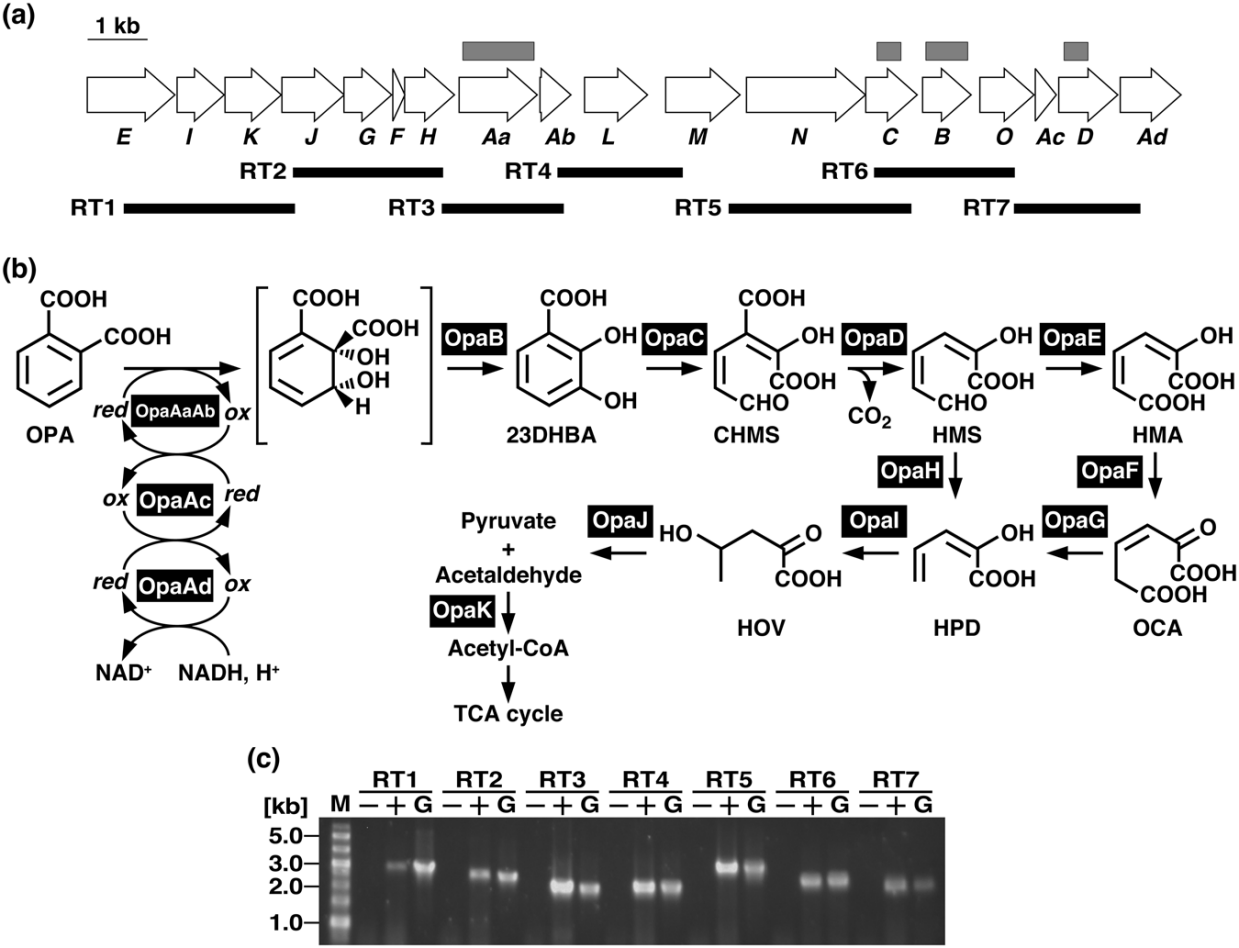
The OPA catabolic pathway genes in *Pseudomonas* sp. strain PTH10. (**a**) Open arrows indicate the sizes, locations, and transcriptional directions of ORFs. The deletion regions of the mutants are indicated by gray boxes above the gene cluster diagram. Boldface bars below the gene cluster diagram indicate the locations of the amplified RT-PCR products shown in panel (c). (**b**) OpaAaAb, large and small subunits, respectively, of oxygenase component of OPA 2,3-dioxygenase; OpaAc, ferredoxin component of OPA 2,3-dioxygenase; OpaAd, ferredoxin reductase component of OPA 2,3-dioxygenase; OpaB, dihydrodiol dehydrogenase; OpaC, 23DHBA 3,4-dioxygenase; OpaD, CHMS decarboxylase; OpaE, HMS dehydrogenase; OpaF, OCA tautomerase; OpaG, OCA decarboxylase; OpaH, HMS hydrolase; OpaI, HPD hydratase; OpaJ, HOV aldolase; and OpaK, acetaldehyde dehydrogenase (acylating). (**c**) The results of agarose gel electrophoresis of RT-PCR products obtained with primers targeting RT1 (expected size 2,865 bp), RT2 (expected size 2,501 bp), RT3 (expected size 2,039 bp), RT4 (expected size 2,100 bp), RT5 (expected size 3,054 bp), RT6 (expected size 2,367 bp), and RT7 (expected size 2,124 bp) are shown. The amplified regions and the primer sequences are indicated in panel (a) and Table S1, respectively. Lanes M, molecular size markers; lanes G, control PCR with the genomic DNA of strain PTH10; lanes + and −, RT-PCR with and without RT, respectively. Because, the image of the gel was cropped, full-length gel image is presented in Supplementary Fig. S4.

The *opaD* gene, thought to encode a decarboxylase, was found in the vicinity of *opaC* (Fig. 1a). As the amino acid sequence of OpaD shares 30% and 28% identity with those of 2-amino-3-carboxymuconate-6-semialdehyde (ACMS) decarboxylase from *P. fluorescens* KU-7 (NbaD; BAC65312) and 5-carboxy-2-hydroxymuconate-6-semialdehyde decarboxylase from *Paenibacillus* sp. strain JJ-1b (PraH; AAU25434), respectively, OpaD might be involved in the decarboxylation of 3-carboxy-2-hydroxymuconate-6-semialdehyde (CHMS) to 2-hydroxymuconate-6-semialdehyde (HMS).

Seven open reading frames (ORFs) including the *opaEIKJGFH* genes, which share identity with those of the aromatic ring *meta*-cleavage pathway, were found in the *opa* gene cluster (Fig. 1a). BLAST analysis showed that these genes encoded a putative HMS dehydrogenase (*opaE*), 2-hydroxypenta-2,4-dienoate (HPD) hydratase (*opaI*), acetaldehyde dehydrogenase (*opaK*), 4-hydroxy-2-oxovalerate (HOV) aldolase (*opaJ*), 4-oxalocrotonate (OCA) decarboxylase (*opaG*), OCA tautomerase (*opaF*), and HMS hydrolase (*opaH*) (Fig. 1a). Based on sequence similarity, these gene products are likely to share the same function (Fig. 1b).

### Characterization of the *opaC* and *opaD* gene products

To determine the enzymatic activity of the *opaC* and *opaD* gene products, each gene was expressed in *E. coli* BL21(DE3). When 100 μM 23DHBA, which has maximum absorption at 240 nm, was incubated with the cell extract containing OpaC at 30 °C, it was converted to a product with maximum absorption at 343 nm (see Fig. S1a in the supplemental material), which is identical to that of CHMS^22^. No conversion was observed when the cell extract of *E. coli* harboring the empty vector was used (data not shown), indicating that 23DHBA was converted to CHMS by the *opaC*-encoding 23DHBA 3,4-dioxygenase.

When the cell extract containing OpaD was added to the resulting mixture from the OpaC reaction, a spectrum with a maximum at 375 nm was detected (see Fig. S1b in the supplemental material). According to previous studies^23,24^, this spectrum is characteristic of HMS, which is produced from CHMS by decarboxylation. As before, conversion of CHMS did not occur in the presence of crude extract from *E. coli* harboring the empty vector (data not shown). These results strongly suggest that *opaD* encodes a CHMS decarboxylase, which catalyzes the decarboxylation of CHMS to HMS.

### Identification of the OPA degradation gene cluster

In the *opa* gene cluster, the genes designated *opaAa* and *opaAb*, appeared to code for the large and small subunits, respectively, of the terminal oxygenase of an aromatic-ring-hydroxylating dioxygenase (Fig. 1 and Table 1). The amino acid sequence of OpaAa showed ca. 30% identity to those of the large subunits of anthranilate 1,2-dioxygenase (AntA) and benzoate 1,2-dioxygenase (BenA) from *Acinetobacter baylyi* ADP1^25^. The amino acid sequence of OpaAb shared 27% and 26% identity with those of the small subunits of anthranilate 1,2-dioxygenase (AntB) and benzoate 1,2-dioxygenase (BenB) from strain ADP1, respectively^25^. Further analysis revealed the existence of two genes named *opaAc* and *opaAd* whose predicted amino acid sequences showed similarity to those of [2Fe-2S]-type ferredoxin and FNR_N_-type reductase, respectively. The amino acid sequence of *opaB*, which was found immediately downstream of *opaC*, showed ca. 50% identity to those of other known dihydrodiol dehydrogenases (Fig. 1a and Table 1). Based on amino acid sequence similarity, OPA seems to be converted to 23DHBA by the sequential reactions of OpaA and OpaB in strain PTH10.

### Expression of genes responsible for the conversion of OPA to 23DHBA

To determine the function of the *opaAaAb*, *opaAc*, *opaAd*, and *opaB* gene products, these genes were individually expressed in *E. coli* BL21(DE3) cells harboring plasmids pCPHAab, pEPHAc, pCPHAd, and pEPHB, respectively. The degradation of 100 μM OPA was assayed with 100 μg each of the crude cell extracts containing OpaAaAb, OpaAc, OpaAd, and OpaB in the presence of 1 mM NADH. When the reaction mixture was analyzed by HPLC, depletion of OPA was accompanied by formation of a compound with a retention time of 2.9 min (see Fig. S2 in the supplemental material). Based on a comparison of the retention time and corresponding absorption spectrum with those of authentic 23DHBA (data not shown), the reaction compound was identified as 23DHBA. These results strongly suggested that OpaA and OpaB were involved in the transformation of OPA to 23DHBA. The amount of OPA decreased in the absence of OpaB, suggesting that the putative dihydrodiol compound was formed from OPA by the OpaA reaction. However, formation of said product could not be detected in this condition. Furthermore, no degradation of OPA was observed in the absence of single OpaA components. These data demonstrate that the terminal oxygenase, OpaAaAb, requires a proper electron transfer system, which could be conveniently provided by the specific ferredoxin and ferredoxin reductase encoded by *opaAc* and *opaAd*, respectively. To investigate substrate specificity, OpaA was incubated with 100 μM terephthalate, isophthalate, and benzoate. No depletion of these compounds was observed, suggesting that OpaA was unable to degrade these phthalate isomers.

### Disruption of the *opa* genes in strain PTH10

**Figure 2:**
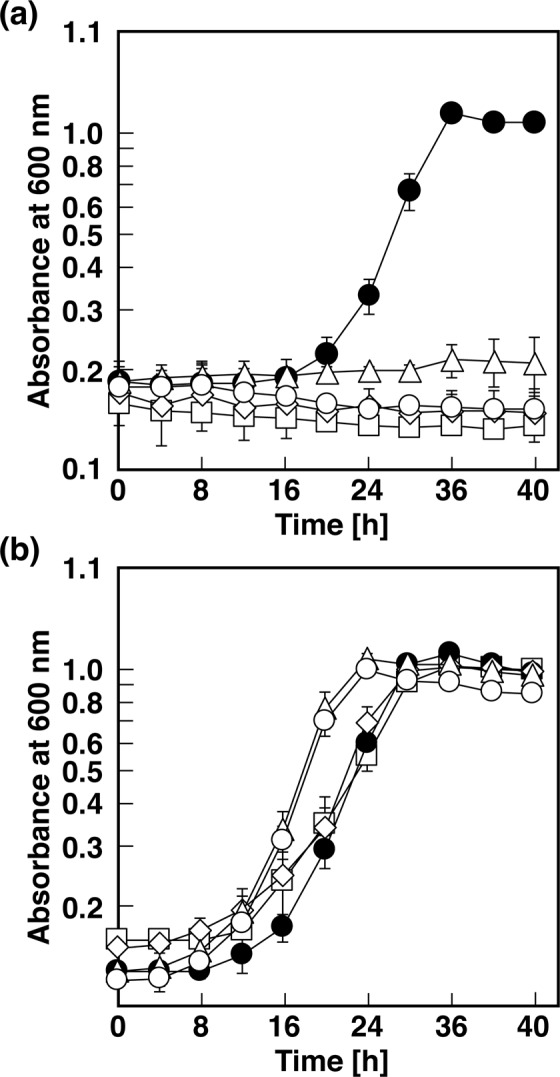
Growth of strain PTH10 and its mutant derivatives on OPA. (**a**) PTH10 (closed circles), DOA (open squares), DOB (open diamonds), DOC (open triangles), and DOD (open circles). (**b**) PTH10 (closed circles), *opaAa*-complementated DOA (open squares), *opaB*-complementated DOB (open diamonds), *opaC*-complementated DOC (open triangles), and *opaD*-complementated DOD (open circles). These strains were grown in W medium containing 10 mM OPA. The data are averages ± standard deviations of three independent experiments performed in parallel.

To investigate the involvement of the *opa* genes in the catabolism of OPA *in vivo*, *opaAa, opaB*, *opaC*, and *opaD* were inactivated by internal deletion using gene replacement. The *opaAa, opaB*, *opaC*, and *opaD* mutants, DOA, DOB, DOC, and DOD, respectively, were unable to grow on OPA as the sole source of carbon and energy (Fig. 2a). To verify that growth deficiency was solely due to the deletion of the *opa* genes, the respective plasmids harboring *opaAa*, *opaB*, *opaC*, or *opaD* in the shuttle vector pJB866 were introduced into the corresponding mutants for complementation. The *opa* genes were expressed using the Pm promoter regulated by XylS in the presence of *m*-toluate, which is not a growth substrate of strain PTH10. Introduction of the plasmids fully restored growth to wild type levels (Fig. 2b). These results indicate that *opaAa, opaB*, *opaC*, and *opaD* are essential for the catabolism of OPA in strain PTH10.

**Figure 3:**
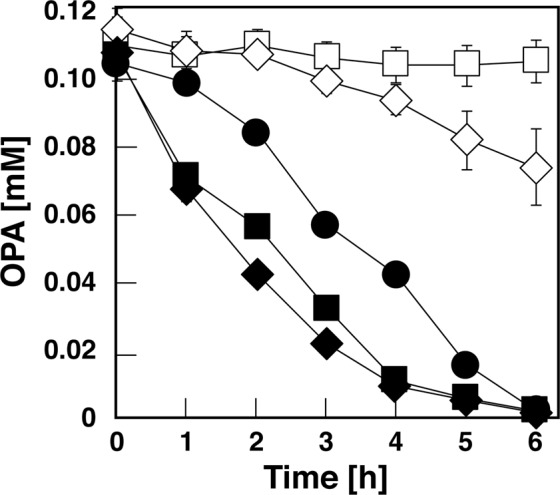
Degradation of OPA by strain PTH10 and its mutant derivatives. The cells of PTH10 (closed circles), DOA (open squares), DOB (open diamonds), *opaAa*-complementated DOA (closed squares), and *opaB*-complementated DOB (closed diamonds) were incubated with 100 μM OPA. The remaining amount of each substrate was determined by HPLC analysis. The values represent the averages ± standard deviations of three independent experiments.

When DOA cells were incubated with 100 μM OPA, the strain completely lost its capacity to catabolize OPA under the assay conditions used (Fig. 3). These results strongly suggest that *opaAa* encodes an oxygenase component of OPA 2,3-dioxygenase (OpaA) and is essential for the hydroxylation of OPA. DOB cells degraded only 20% of OPA after 6 h of incubation, whereas strain PTH10 degraded all the OPA. When the respective plasmids carrying *opaAa* or *opaB* were introduced into the corresponding mutants, their degradation activity was restored to wild type levels (Fig. 3). This finding indicates that the lack of utilization is due to the inactivated gene and is not caused by a polar effect.

**Figure 4:**
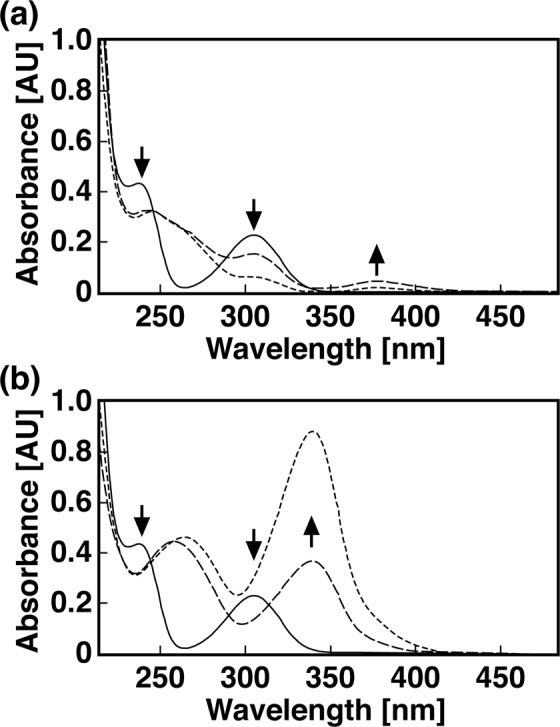
Conversion of 23DHBA by strain PTH10 and its mutant derivative. The reaction mixtures containing 100 μM 23DHBA and the cells of PTH10 (**a**) and DOD (**b**) were incubated at 30 °C. UV-visible spectra were recorded at the start, after 3, and 6 hours of incubation, which are represented by the solid, dashed, and dotted lines, respectively.

DOC cells degraded OPA at the same rate as the wild type, leading to accumulation of an almost identical amount of 23DHBA (see Fig. S3 in the supplemental material). Furthermore, this strain lost completely its ability to degrade 23DHBA, indicating that the *opaC*-dependent 23DHBA *meta*-cleavage pathway was essential for OPA degradation. When DOD cells were incubated with OPA or 23DHBA, these compounds were depleted to the same extent as when the wild type strain was used (see Fig. S3 in the supplemental material). However, accumulation of CHMS (λ_max_ = 343 nm) was observed during the degradation of 23DHBA (Fig. 4), indicating that *opaD* was required for the decarboxylation of CHMS in strain PTH10.

### Transcriptional analysis of the *opaAa* gene

To identify the operon structure of the *opa* genes, reverse transcription PCR (RT-PCR) analysis was performed with total RNA extracted from strain PTH10 grown on OPA as the sole carbon and energy source. RT-PCR amplification products of the expected size were detected for the intergenic regions of *opaE*-*opaJ*, *opaJ*-*opaH*, *opaH*-*opaAb*, *opaAb*-*opaM*, *opaM*-*opaC*, *opaC*-*opaO*, and *opaO*-*opaAd* (Fig. 1b,c). These results suggest that the *opa* genes are organized in the same transcriptional unit.

**Figure 5:**
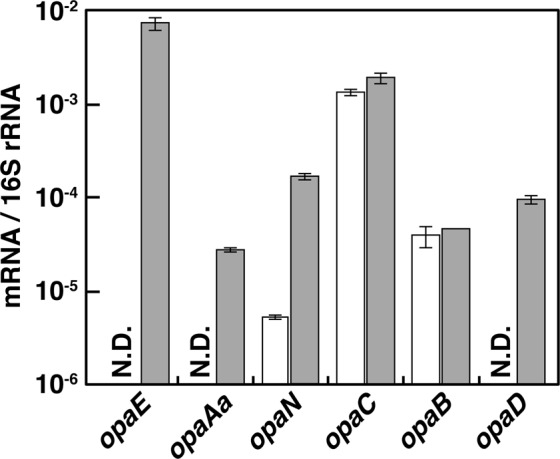
Quantification of the expression levels of the *opa* genes. Total RNA was isolated from the cells of PTH10 grown in W medium containing 10 mM succinate (open bars) or 10 mM OPA (grey bars). The mRNA expression levels were calculated as a ratio of 16S rRNA gene expression. The data are mean values ± standard deviations for three independent experiments. N.D.: Not detected.

To determine whether transcription of the *opa* operon was induced in response to OPA, mRNA levels of *opaAa*, *opaB*, *opaC*, *opaD*, *opaE*, and *opaN* were measured by quantitative reverse transcription-PCR (qRT-PCR) analysis using the total RNA harvested from the cells grown on OPA or succinate. When the PTH10 cells grew on succinate, mRNAs of *opaAa*, *opaD*, and *opaE* were not detected in our analytical condition (Fig. 5). The transcription of *opaN* was extremely low level. On the other hand, these genes transcription was significantly increased in the cells grown on OPA, suggesting that the transcription of these *opa* genes is induced during OPA utilization. At the same time, the *opaB* and *opaC* genes were constitutively expressed at a high level under non-induced conditions (Fig. 5). This suggests the possible existence of an additional internal promoter for the constitutive expressions of *opaB* and *opaC*. Furthermore, transcription of *opaD* located downstream of *opaB* was induced in the presence of OPA, suggesting that an additional inducible promoter is available to control also the *opaD* transcription.

## Discussion

The present study reports the identification of the *opa* gene cluster, which is involved in the OPA catabolic pathway in *Pseudomonas* sp. strain PTH10. Degradation via 23DHBA is notably different from other known OPA catabolic pathways^12,14^. Although, the 23DHBA *meta*-cleavage pathway has been found in *P. reinekei* MT1^22^, growth ability on OPA and the genes responsible for its conversion to 23DHBA have never been reported. The amino acid sequence of OpaC presents similarity to that of DhbA from MT1, which belongs to the type I extradiol dioxygenase family^26^. In the presence of either OpaC or DhbA, CHMS was generated as a reaction product from 23DHBA, suggesting that these enzymes share a similar reaction mechanism for the aromatic ring cleavage of 23DHBA. The *dhbB* gene-encoding class II aldolase is involved in the decarboxylation of CHMS in MT1^22^. In contrast, the CHMS decarboxylation of strain PTH10 requires OpaD, which belongs to a metallo-dependent hydrolase superfamily^27^. Crystallographic analysis of NbaD has revealed that the active site Zn ion is directly bound to His9, His11, His177, Asp294, and a water molecule that is coordinated by hydrogen bonds with His228 and Asp294^28^. Given that the corresponding amino acid residues of OpaD are well conserved, they might be involved in the coordination of this enzyme’s active site.

Deduced amino acid sequence similarities of *opaAaAbAcAd* revealed that OpaA belonged to the type III Rieske non-heme iron oxygenase family^29^. Amino acid sequence alignment between OpaAa and the oxygenase large subunit of naphthalene 1,2-dioxygenase from *Pseudomonas* sp. NCIB 9816-4, which belongs to the same family of protein^30^, revealed high conservation of the amino acid residues responsible for the binding of mononuclear non-heme iron (His208, His213, and Asp362) and Rieske-type [2Fe-2S] centers (Cys81, Cys101, His83, and His104) are well conserved^30,31^. We speculate that the corresponding residues in OpaAa act as ligands for mononuclear non-heme iron and as an [2Fe-2S] cluster.

As shown in Fig. 3, OPA degradation was affected by deletion of *opaB*, opening the possibility that OPA hydroxylation was inhibited by accumulation of the dihydrodiol compound in this strain. Transcription of *opaB* might be driven by a constitutive promoter located in the internal region of the *opa* operon. A similar case has been reported for the 2-chloronitrobenzene degradation gene cluster of *P. stutzeri* ZWLR2-1^32^. There, the internal promoter enhances gene expression to optimize the degradation of 2-chloronitrobenzene. In the case of PTH10, the constitutive expression of *opaB* and *opaC* is most likely required for OPA catabolism, as it allows cells to prevent the accumulation of the dihydrodiol and diol compounds and utilize the substrate efficiently.

According to their sequence similarity and the transcriptional induction during the growth of PTH10 on OPA (Fig. 5), the *opaEIKJGFH* gene products might be involved in the lower branch of the OPA catabolism pathway (Fig. 1a). Organization of the *opaEIKJGFH* genes is almost identical to that of the catechol *meta*-cleavage pathway genes of *P. putida* mt-2 (*xyl*; accession number AJ344068)^33,34^ and *Pseudomonas* sp. CF600 (*dmp*; accession numbers M33263, X52805, and X60835)^35–37^. In the catechol *meta*-cleavage pathway gene clusters, the HMS hydrolase genes (*xylF* and *dmpD*) are located between the HMS dehydrogenase genes (*xylG* and *dmpC*) and HPD hydratase genes (*xylJ* and *dmpE*). However, in strain PTH10, the HMS hydrolase gene (*opaH*) was located in the terminal region of this cluster. Furthermore, the gene corresponding to *xylT* encoding a chloroplast-type ferredoxin was not observed in the *opa* gene cluster.

The amino acid sequence of *opaL* and *opaN* products showed similarity to a solute-binding protein (SBP) and an integral membrane protein, respectively, of tripartite ATP-independent periplasmic (TRAP) transporter^38^. TRAP transporter is implicated in the transport of aromatic compounds and has been characterized in *Comamonas* sp. DJ-12^39^, *Aromatoleum aromaticum* EbN1^40^, and *Rhodopseudomonas palustris*^41^. These strains have been reported to have a SBP belonging to the DctP family^42^, and small and large integral membrane proteins related to DctQ and DctM, respectively, which are involved in the uptake of aromatic compounds. In contrast, the *opaL* gene product is related to another family of SBP, the TRAP-associated extracytoplasmic immunogenic (TAXI) protein family^43^. The TAXI-TRAP system, which contains a TAXI-SBP and a DctQ-DctM fused integral membrane protein, has been invariably found in Archaea^43^. Because the primary structure of OpaN consists of contiguous segments corresponding to DctQ and DctM, which are joined to form a single protein, *opaL* and *opaN* seemed to code for the TAXI-TRAP system for the uptake of OPA. However, to our knowledge, no functional characterization of the TAXI-TRAP system has been reported. Therefore, further characterization of these genes will be required to gain a better understanding of the transport of OPA by the TAXI-TRAP system.

Transcriptional analysis revealed that the *opa* genes consisted of a single transcriptional unit induced during OPA utilization. The transcription of the *opa* genes was thought to be regulated by an unknown transcriptional regulator. However, no genes encoding such a transcriptional regulator were found in this gene cluster. Instead, the constitutive expression of *opaB* and *opaC* and the transcriptional induction of *opaD* located downstream of *opaB*, suggest the presence of several internal promoters in the *opa* operon, which are involved in the transcription of these genes. Unfortunately, the consensus promoter sequence could not be found in the upstream region of these *opa* genes. Further studies must be conducted to elucidate the transcriptional regulation of the *opa* operon.

## Methods

### Bacterial strains, plasmids, and culture conditions

*Pseudomonas* sp. strain PTH10 and its mutant derivatives were routinely grown at 30 °C in Luria-Bertani (LB), 1/5 LB, or W minimal salt medium^44^ containing 10 mM OPA or 10 mM succinate. *Escherichia coli* strains were grown in LB medium at 30 °C or 37 °C. If necessary, ampicillin, chloramphenicol, tetracycline, and kanamycin were added into the medium at the concentrations of 100, 20, 10, and 50 mg/l, respectively. For enrichment culture, 100 ml of W minimal salt medium containing 10 g of soil and 1 mM OPA was incubated at 30 °C for a week.

### DNA manipulations, sequencing, and analysis

DNA manipulations including chromosomal and plasmid DNA isolation, electroporation, and nucleotide sequencing were performed according to methods described previously^45–47^. Analyses of nucleotide and amino acid sequences were carried out as previously described^48^.

The draft genome sequence of strain PTH10 was generated using the 454 GS-FLX Titanium (Roche, Basel, Switzerland) and the Illumina HiSeq 2000 system (Illumina, San Diego, CA, USA). The reads from these two systems were assembled by using Newbler version 2.6 (Roche). The assembly contains 40 contigs with a total genome size of 4,184,609 bp (*N*_50_ of 199,694 bp; G + C content of 63.2%). The genome sequence was annotated using RAST server^49^.

### Analysis of disruption mutants

To construct the *opa* gene disruption mutants, the *opaAa*, *opaB*, *opaC*, and *opaD* genes were disrupted by in-flame deletion of their central region. The fragments containing the N- and the C-terminal sequences were ligated and inserted in pK18*mobsacB*^50^. Each of the resulting plasmid was introduced into the cells of PTH10 by electroporation, and candidates for the deletion mutants were isolated as described previously^51^. Disruption of the genes was confirmed by diagnostic PCR using specific primer sets and subsequently by DNA sequencing of the PCR amplified regions flanking the deletions.

To construct the complementary plasmids, the specific primer pairs (Table S1) were used to amplify DNA fragments spanning each *opa* gene. The amplified fragments were cloned into the shuttle vector pJB866^52^ using In-Fusion HD cloning kit (Clontech, CA, USA). The resulting plasmids carrying *opaAa*, *opaB*, *opaC*, or *opaD* were independently introduced into the corresponding mutant cells by electroporation for complementation studies. The plasmid maintained in each cell was confirmed by the plasmid extraction form the resulting transformants and PCR amplification of the corresponding gene regions.

To determine the degradation ability of PTH10 and its mutant derivatives toward OPA or 23DHBA, the cells were grown on LB medium to mid-logarithmic phase. Then, the cells were corrected by the centrifugation and inoculated into 1-ml W minimal salt medium containing 1 mM OPA or 23DHBA. Then the cells were incubated at 30 °C, and the concentration of these substrates were measured by HPLC analysis.

### Expression of the *opa* genes in *E. coli*

The *opaAaAb*, *opaAd*, and *opaD* genes were independently PCR amplified using the specific primer pairs (Table S1) and ligated into the expression vector pColdIV using In-Fusion HD cloning kit to construct pCPHAab, pCPHAd, and pCPHD, respectively. The *opaB* and *opaC* genes were independently PCR amplified and ligated into pET21a using In-Fusion HD cloning kit to construct pEPHB and pEPHC, respectively. The *opaAc* gene was PCR amplified and cloned into pET16b by In-Fusion cloning to construct pEHTAc. The transformants of *E. coli* BL21(DE3) harboring the pColdIV, pET21a, and pET16b derivatives were grown at 30 °C. Expressions of the *opaAc*, *opaB*, and *opaC* were induced for 4 h by adding 1 mM isopropyl-β-D-thiogalactopyranoside (IPTG) when the absorbance of the culture at 600 nm reached 0.5. The *opaAaAb*, *opaAd*, and *opaD* expressions were induced by cultivation at 15 °C for 24 h after the addition of 1 mM IPTG. After the induction of the gene expression, the cells were harvested by centrifugation at 5,000 × *g* for 10 min and resuspended in 50 mM Tris-HCl buffer (pH 7.5). Then they were disrupted by an ultrasonic disintegrator (UD-201; Tomy Seiko Co., Tokyo, Japan) and centrifuged at 15,000 × *g* for 15 min. The resulting supernatants were used as crude enzymes.

### Enzymatic assays

Conversion of OPA to 23DHBA was monitored by HPLC analysis. The 1-ml assay mixture consisted of 50 mM Tris-HCl buffer (pH 7.5), 100 μM OPA, 1 mM NADH, and the cell extracts containing OpaAaAb, OpaAc, OpaAd, and OpaB (100 μg of protein each) was incubated at 30 °C. A portion of the reaction mixture was removed at various sampling times and analyzed using HPLC.

23DHBA 3,4-dioxygenase activity was monitored using a DU-800 spectrophotometer (Beckman coulter, Fullerton, Calif.). The reaction mixture (final volume, 1 ml) containing 50 mM Tris-HCl buffer (pH 7.5), 100 μM 23DHBA, and the cell extract of BL21(DE3) harboring pEPHC (10 μg of protein) was preincubated without the substrate for 1 min at 30 °C, and then the reaction was started by adding 23DHBA. 23DHBA 3,4-dioxygenase activity was also assayed by measuring the substrate-dependent oxygen consumption rate. A 2-ml assay mixture contained 50 mM GTA buffer (pH 7.5) consisting of 50 mM 3,3-dimethylglutarate, 50 mM Tris, and 50 mM 2-amino-2-methyl-1,3-propanediol, the cell extract (10 µg of protein), and 1 mM 23DHBA. The reaction mixture was incubated at 30 °C. The oxygen consumption rate was determined with an oxygen electrode (B-505; Iijima Electronics Manufacturing Co., Ltd., Aichi, Japan). One unit of enzyme activity was defined as the amount of enzyme that resulted in consumption of 1 µmol of O_2_ per 1 min at 30 °C. Specific activity was expressed in units per milligram of protein.

The CHMS decarboxylase activity was assayed spectrophotometrically by monitoring the production of HMS from CHMS using a preassay mixture that consisted of 100 μM 23DHBA and the cell extract including OpaC (10 μg of protein) in 50 mM Tris-HCl buffer (pH 7.5) in a total volume of 900 μl. The mixture was incubated for 5 min at 30 °C. After the reaction was completed, the cell extract of BL21(DE3) harboring pCPHD and pG-Tf2 (the crude OpaD; 10 μg of protein) was added in a final volume of 1 ml, and then the decrease in the absorbance at 343 nm and the increase in the absorbance at 375 nm were monitored at 30 °C.

### Analytical methods

The protein concentration was determined by the method of Bradford^53^. The sizes of the proteins expressed in *E. coli* were determined by sodium dodecyl sulfate-12% polyacrylamide gel electrophoresis (SDS-PAGE). The proteins in the gels were stained with Coomassie brilliant blue R-250. HPLC analysis was performed with 1290 Infinity LC (Agilent technologies, Santa Clara, CA) equipped with a HC-C18(2) column (150 mm by 4.6 mm; Agilent technologies). The mobile phase was a mixture of water (74.5%), acetonitrile (24.5%), and phosphoric acid (1%) at a flow rate of 1.5 ml/min. The retention times of OPA and 23DHBA were 2.3 and 2.9 min, respectively.

### RT-PCR and qRT-PCR analyses

The cells of PTH10 were grown in W minimal salt medium supplemented with 10 mM OPA or succinate at 30 °C. When the absorbance of the culture at 600 nm reached approximately 0.8, the cells were harvested by centrifugation at 5,000 × *g* at 4 °C for 10 min. Total RNA was isolated with NucleoSpin RNA Plus (Macherey-Nagel, Düren, Germany) and treated with TURBO DNase (Thermo Fisher Scientific, Waltham, MA, USA). Single-stranded cDNA was synthesized from 1.0 μg of total RNA with 200 U of PrimeScript II reverse transcriptase (Takara Bio, Inc., Shiga, Japan) and random 6-mer primers in a 20-μl reaction mixture. The cDNA mixture of 1.0 μl was used to perform PCR amplification in a 25-μl mixture using specific primers (Table S1) and 0.65 U of PrimeStar GXL DNA polymerase (TaKaRa Bio Inc., Otsu, Japan) under the following conditions: 98 °C for 5 min plus 25 cycles of 98 °C for 10 s, 57 °C for 15 s, and 68 °C for 30 s. A control without reverse transcriptase was used for each reaction to verify the absence of genomic DNA contamination. PCR-amplified samples were electrophoresed on a 0.8% agarose gel and visualized with ethidium bromide. qRT-PCR analysis was carried out in a 25-μl mixture using the specific primers (Table S1) according to the previous report^54^.

### Nucleotide sequence accession number

The nucleotide sequence of 16S rRNA gene and the *opa* genes reported in this paper have been deposited in the DDBJ, EMBL, and GenBank nucleotide sequence databases under accession number LC427665 and LC314585, respectively.

### Accession Code

The nucleotide sequence reported in this paper has been deposited in the DDBJ, EMBL, and GenBank nucleotide sequence databases under accession number LC314585.

## Supplementary information

Figure S1, S2, S3, S4, Table S1

## References

[1] Nakai M (1999). Binding characteristics of dialkyl phthalates for the estrogen receptor. Biochem Biophys Res Commun.

[2] Harris CA, Henttu P, Parker MG, Sumpter JP (1997). The estrogenic activity of phthalate esters *in vitro*. Environ Health Perspect.

[3] Ganning AE, Brunk U, Dallner G (1984). Phthalate esters and their effect on the liver. Hepatology.

[4] Giam CS, Chan HS, Neff GS, Atlas EL (1978). Phthalate ester plasticizers: a new class of marine pollutant. Science.

[5] Vamsee-Krishna C, Phale PS (2008). Bacterial degradation of phthalate isomers and their esters. Indian J Microbiol.

[6] Hara H, Stewart GR, Mohn WW (2010). Involvement of a novel ABC transporter and monoalkyl phthalate ester hydrolase in phthalate ester catabolism by *Rhodococcus jostii* RHA1. Appl Environ Microbiol.

[7] Sepic E, Bricelj M, Leskovsek H (1998). Degradation of fluoranthene by *Pasteurella* sp. IFA and *Mycobacterium* sp. PYR-1:isolation and identification of metabolites. J Appl Microbiol.

[8] Grifoll M, Selifonov SA, Chapman PJ (1994). Evidence for a novel pathway in the degradation of fluorene by *Pseudomonas* sp. strain F274. Appl Environ Microbiol.

[9] Barnsley EA (1983). Bacterial oxidation of naphthalene and phenanthrene. J Bacteriol.

[10] Chang HK, Zylstra GJ (1998). Novel organization of the genes for phthalate degradation from *Burkholderia cepacia* DBO1. J Bacteriol.

[11] Pujar BG, Ribbons DW (1985). Phthalate metabolism in *Pseudomonas fluorescens* PHK: purification and properties of 4,5-dihydroxyphthalate decarboxylase. Appl Environ Microbiol.

[12] Nakazawa T, Hayashi E (1977). Phthalate metabolism in *Pseudomonas testosteroni*: accumulation of 4,5-dihydroxyphthalate by a mutant strain. J Bacteriol.

[13] Stingley RL, Brezna B, Khan AA, Cerniglia CE (2004). Novel organization of genes in a phthalate degradation operon of *Mycobacterium vanbaalenii* PYR-1. Microbiology.

[14] Eaton RW (2001). Plasmid-encoded phthalate catabolic pathway in *Arthrobacter keyseri* 12B. J Bacteriol.

[15] Habe H (2003). Phthalate catabolic gene cluster is linked to the angular dioxygenase gene in *Terrabacter* sp. strain DBF63. Appl Environ Microbiol.

[16] Patrauchan MA (2005). Catabolism of benzoate and phthalate in *Rhodococcus* sp. strain RHA1: redundancies and convergence. J Bacteriol.

[17] Fukuhara Y (2010). Characterization of the isophthalate degradation genes of *Comamonas* sp. strain E6. Appl. Environ. Microbiol..

[18] Schläfli HR, Weiss MA, Leisinger T, Cook AM (1994). Terephthalate 1,2-dioxygenase system from *Comamonas testosteroni* T-2: purification and some properties of the oxygenase component. J. Bacteriol..

[19] Choi KY (2005). Molecular and biochemical analysis of phthalate and terephthalate degradation by *Rhodococcus* sp. strain DK17. FEMS Microbiol. Lett..

[20] Wang YZ, Zhou Y, Zylstra GJ (1995). Molecular analysis of isophthalate and terephthalate degradation by *Comamonas testosteroni* YZW-D. Environ. Health Perspect..

[21] Sasoh M (2006). Characterization of the terephthalate degradation genes of *Comamonas* sp. strain E6. Appl. Environ. Microbiol..

[22] Marín M, Plumeier I, Pieper DH (2012). Degradation of 2,3-dihydroxybenzoate by a novel *meta*-cleavage pathway. J Bacteriol.

[23] Crawford RL, Bromley JW, Perkins-Olson PE (1979). Catabolism of protocatechuate by *Bacillus macerans*. Appl Environ Microbiol.

[24] Sala-Trepat JM, Evans WC (1971). The *meta* cleavage of catechol by *Azotobacter* species. 4-Oxalocrotonate pathway. Eur J Biochem.

[25] Bundy BM, Campbell AL, Neidle EL (1998). Similarities between the *antABC*-encoded anthranilate dioxygenase and the *benABC*-encoded benzoate dioxygenase of *Acinetobacter* sp. strain ADP1. J Bacteriol.

[26] Vaillancourt FH, Bolin JT, Eltis LD (2006). The ins and outs of ring-cleaving dioxygenases. Crit Rev Biochem Mol Biol.

[27] Liu A, Zhang H (2006). Transition metal-catalyzed nonoxidative decarboxylation reactions. Biochemistry.

[28] Martynowski D (2006). Crystal structure of α-amino-β-carboxymuconate-ε-semialdehyde decarboxylase: insight into the active site and catalytic mechanism of a novel decarboxylation reaction. Biochemistry.

[29] Kweon O (2008). A new classification system for bacterial Rieske non-heme iron aromatic ring-hydroxylating oxygenases. BMC biochemistry.

[30] Kauppi B (1998). Structure of an aromatic-ring-hydroxylating dioxygenase-naphthalene 1,2-dioxygenase. Structure.

[31] Parales RE (2003). The role of active-site residues in naphthalene dioxygenase. J Ind Microbiol Biotechnol.

[32] Gao YZ, Liu H, Chao HJ, Zhou NY (2016). Constitutive expression of a Nag-like dioxygenase gene through an internal promoter in the 2-chloronitrobenzene catabolism gene cluster of *Pseudomonas stutzeri* ZWLR2-1. Appl Environ Microbiol.

[33] Franklin FC, Bagdasarian M, Bagdasarian MM, Timmis KN (1981). Molecular and functional analysis of the TOL plasmid pWWO from *Pseudomonas putida* and cloning of genes for the entire regulated aromatic ring *meta* cleavage pathway. Proc Natl Acad Sci USA.

[34] Harayama S, Lehrbach PR, Timmis KN (1984). Transposon mutagenesis analysis of *meta*-cleavage pathway operon genes of the TOL plasmid of *Pseudomonas putida* mt-2. J Bacteriol.

[35] Nordlund I, Shingler V (1990). Nucleotide sequences of the *meta*-cleavage pathway enzymes 2-hydroxymuconic semialdehyde dehydrogenase and 2-hydroxymuconic semialdehyde hydrolase from *Pseudomonas* CF600. Biochim Biophys Acta.

[36] Bartilson M, Shingler V (1989). Nucleotide sequence and expression of the catechol 2,3-dioxygenase-encoding gene of phenol-catabolizing *Pseudomonas* CF600. Gene.

[37] Shingler V, Powlowski J, Marklund U (1992). Nucleotide sequence and functional analysis of the complete phenol/3,4-dimethylphenol catabolic pathway of *Pseudomonas* sp. strain CF600. J Bacteriol.

[38] Mulligan C, Fischer M, Thomas GH (2011). Tripartite ATP-independent periplasmic (TRAP) transporters in bacteria and archaea. FEMS Microbiol Rev.

[39] Chae JC, Kim Y, Kim YC, Zylstra GJ, Kim CK (2000). Genetic structure and functional implication of the *fcb* gene cluster for hydrolytic dechlorination of 4-chlorobenzoate from *Pseudomonas* sp. DJ-12. Gene.

[40] Trautwein K (2012). Benzoate mediates repression of C_4_-dicarboxylate utilization in “*Aromatoleum aromaticum*” EbN1. J Bacteriol.

[41] Salmon RC, Cliff MJ, Rafferty JB, Kelly DJ (2013). The CouPSTU and TarPQM transporters in *Rhodopseudomonas palustris*: redundant, promiscuous uptake systems for lignin-derived aromatic substrates. PloS one.

[42] Shaw JG, Hamblin MJ, Kelly DJ (1991). Purification, characterization and nucleotide sequence of the periplasmic C_4_-dicarboxylate-binding protein (DctP) from *Rhodobacter capsulatus*. Mol Microbiol.

[43] Kelly DJ, Thomas GH (2001). The tripartite ATP-independent periplasmic (TRAP) transporters of bacteria and archaea. FEMS Microbiol Rev.

[44] Araki N (2011). Identification and characterization of uptake systems for glucose and fructose in *Rhodococcus jostii* RHA1. J Mol Microb Biotech.

[45] Abe T, Masai E, Miyauchi K, Katayama Y, Fukuda M (2005). A tetrahydrofolate-dependent *O*-demethylase, LigM, is crucial for catabolism of vanillate and syringate in *Sphingomonas paucimobilis* SYK-6. J Bacteriol.

[46] Ausubel, F. M. *et al*. *Current protocols in molecular biology*. (John Wiley & Sons, Inc., 1990).

[47] Sambrook, J., Fritsch, E. F. & Maniatis, T. *Molecular cloning: a laboratory manual, 2nd ed*., (Cold Spring Harbor Laboratory Press, 1989).

[48] Kasai D (2009). Uncovering the protocatechuate 2,3-cleavage pathway genes. J Bacteriol.

[49] Aziz, R. K. *et al*. The RAST Server: rapid annotations using subsystems technology. *BMC Genomics*, 10.1186/1471-2164-9-75 (2008). 10.1186/1471-2164-9-75 PMC2265698 18261238

[50] Schäfer A (1994). Small mobilizable multi-purpose cloning vectors derived from the *Escherichia coli* plasmids pK18 and pK19: selection of defined deletions in the chromosome of *Corynebacterium glutamicum*. Gene.

[51] Masai E (1999). Genetic and biochemical characterization of a 2-pyrone-4,6-dicarboxylic acid hydrolase involved in the protocatechuate 4,5-cleavage pathway of *Sphingomonas paucimobilis* SYK-6. J Bacteriol.

[52] Blatny JM, Brautaset T, Winther-Larsen HC, Karunakaran P, Valla S (1997). Improved broad-host-range RK2 vectors useful for high and low regulated gene expression levels in gram-negative bacteria. Plasmid.

[53] Bradford MM (1976). A rapid and sensitive method for the quantitation of microgram quantities of protein utilizing the principle of protein-dye binding. Anal Biochem.

[54] Kasai D, Kitajima M, Fukuda M, Masai E (2010). Transcriptional regulation of the terephthalate catabolism operon in *Comamonas* sp. strain E6. Appl Environ Microbiol.

