# Supplementary material for: 2,3-Dihydroxybenzoate meta-Cleavage Pathway is Involved in o-Phthalate Utilization in Pseudomonas sp. strain PTH10
Source: Sci Rep. 2019 Feb 4;9:1253. doi: 10.1038/s41598-018-38077-2 (PMC6362003; doi:10.1038/s41598-018-38077-2)
Supplement: Supplementary file 1 — Figure S1, S2, S3, S4, Table S1 [file 41598_2018_38077_MOESM1_ESM.pdf]

2,3-Dihydroxybenzoate *meta*-Cleavage Pathway Is Involved in *o*-Phthalate Utilization in *Pseudomonas* sp. strain PTH10

Daisuke Kasai,[1#] Takumi Iwasaki,[2] Kazuki Nagai,[1] Naoto Araki,[1] Tatsunari Nishi,[2] and Masao Fukuda[1$]

Department of Bioengineering, Nagaoka University of Technology, Nagaoka, Niigata, 940-2188,[1] and Genaris, Inc., Yokohama, Kanagawa, 230-0046,[2] Japan

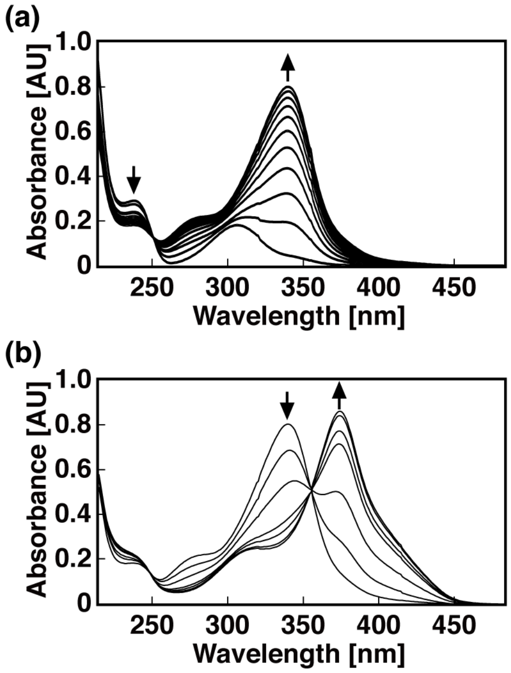

Fig. S1

Fig. S1. Conversion of 23DHBA and CHMS by the cell extract of *E. coli* BL21(DE3) expressed the *opaC* and *opaD* genes, respectively. (a) A reaction mixture containing 100 μM 23DHBA and the cell extract of *E. coli* expressed *opaC* (10 μg of protein) was incubated at 30°C. UV-visible spectra were recorded for 10 min at 1-min intervals. (b) The OpaC reaction mixture containing the cell extract of *E. coli* expressed *opaD* (10 μg of protein) was incubated at 30°C. UV-visible spectra were recorded for 6 min at 1-min intervals.

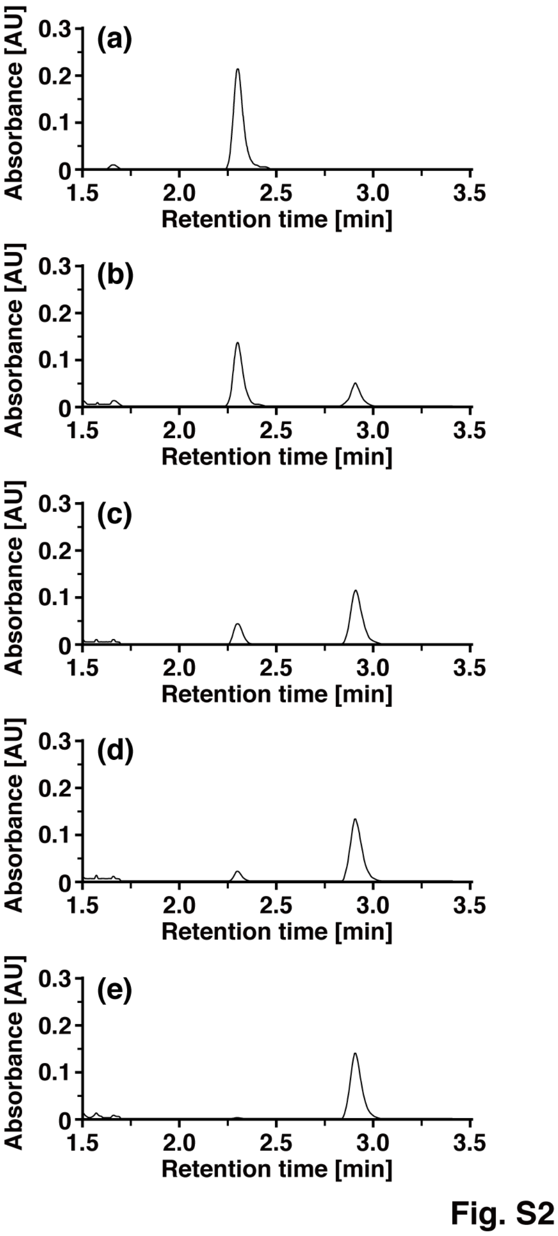

Fig. S2. Conversion of OPA to 23DHBA catalyzed by OpaA and OpaB. The reaction mixture consisting 100 μM OPA, 1 mM NADH, and the cell extracts of *E. coli* expressed *opaAaAb*, *opaAc*, *opaAd*, and *opaB* (100 μg of protein each) in 50 mM Tris-HCl buffer (pH 7.5) in a total volume of 1 ml was incubated at 30°C. Panels (a) to (e) showed HPLC chromatograms of the reaction mixtures at the start and after 30, 60, 180, and 360 min of incubation. Compounds were monitored at 230 nm.

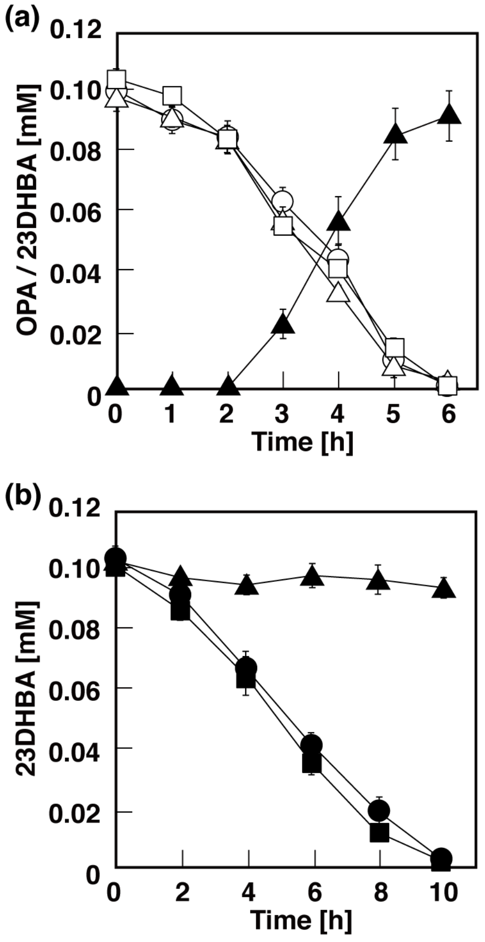

Fig. S3

Fig. S3. Degradation of OPA (a) and 23DHBA (b) by strain PTH10 and its mutant derivatives. Resting cells of PTH10 (squares), DOC (triangles), and DOD (circles) were incubated with 100 μM OPA or 23DHBA. The remaining amount of each substrate was determined by HPLC analysis. The amount of OPA and 23DHBA were showed by open and closed symbols, respectively. The values represent the averages ± standard deviations of three independent experiments.

Table S1 Primer sequences used in this study

Primers	Sequences (5′-3′)
RT-PCR analysis	
RT1_F	TCTACAACGTGGTGCATGGT
RT1_R	GACCGCTTCTATCCATTCCA
RT2_F	GGAATGGATAGAAGCGGTCA
RT2_R	GATCAGGGTTTCCTTGTCCA
RT3_F	GCTGACCTCGAAGAAGCTGT
RT3_R	CGTTGTTCAGCAGCATCACT
RT4_F	GAACATCTGGTTCGGCAAAT
RT4_R	ACTGATTGCCCAGGTACAGG
RT5_F	AGATCGACATGCTGATGCTG
RT5_R	ACGATGGCTTCATCTCGAAC
RT6_F	GCGCCTTCAATCTCTGCTAC
RT6_R	AAAACAACCAGGACGAGGTG
RT7_F	CACCTCGTCCTGGTTGTTTT
RT7_R	GAAACGGAACTCGGTGATGT
qRT-PCR analysis	
qPCR_opaAa_F	AGCGAGATTCCCAGGTTCGG
qPCR_opaAa_R	GGCAGCGGTTGATCAGCAAA
qPCR_opaB_F	AACCGTTCTGGCATTACAGC
qPCR_opaB_R	AATAGGGGATGCGGAAAATC
qPCR_opaD_F	CAGCACGGTTGGGAAATTAG
qPCR_opaD_R	AGACCAGGGTGTCGTACCAG
qPCR_opaC_F	GACCTGCATTTCTGGACCTC
qPCR_opaC_R	GTGATGCACCTCGTCGATAC
qPCR_opaE_F	TTTTCGAAACCTTCGTCCAG
qPCR_opaE_R	TCGTAATAGGCGAGCACCTT
qPCR_opaN_F	ATCGGCGACTACATCCTGTC
qPCR_opaN_R	TCGTGGTAGCGACTCATCTG
qPCR_16s_F	GTGCAAGCGTTAATCGGAAT
qPCR_16s_R	GAAAGCAGTTCCCAGGTTGA
Heterologous expression	
INF_opaAab_F	AAGGAGATATACATATGACAACGCTGATTGCATCC
INF_opaAab_R	GTCATGCTAGCCATATCAGACCAAGAAGGTCAGG
INF_opaAc_F	AAGGAGATATACATATGTCCCGCTTCTACGTCTGC
INF_opaAc_R	GTCATGCTAGCCATACTAGGGCTTGACTTCACCGG
INF_opaAd_F	AAGGAGATATACATATGCCGATCGTACATATCC
INF_opaAd_R	GTCATGCTAGCCATATCCTCGTCGGGCGCCGTACC
INF_opaB_F	AAGGAGATATACATATGAACGAACACAACTGC
INF_opaB_R	GTCATGCTAGCCATATCAGCGGTTCACGGACGAGATGC
INF_opaC_F	AAGGAGATATACATATGATCAATCTGCACGATATCTGC
INF_opaC_R	GTCATGCTAGCCATATTCGCTCACTCGCTGAATTCC
INF_opaD_F	AAGGAGATATACATATGATCGATATGCATACCC
INF_opaD_R	GTCATGCTAGCCATATTATCGGATTGAGCGCAACGG
Gene deletion mutants	
INF_opaAa_UP_F	CCATGATTACGAATTATTCGCGCTACGAGAACTTCAGG
INF_opaAa_UP_R	TTGGGTGATTTCATCCACGCGGTCCTCCTTGACCAGTGC
INF_opaAa_DW_F	GATGAAATCACCCAACGGGCGCAG
INF_opaAa_DW_R	TACCGAGCTCGAATTGCTCAAGGTCTATGCGCGCAAGC
INF_opaB_UP_F	CCATGATTACGAATTATTCGCTGGTCGCCGTGATGGTGG
INF_opaB_UP_R	CACCTCACCGGTGATCGCCACCTTGCCGACGAATCGTCTGC
INF_opaB_DW_F	ATCACCGGTGAGGTGCTGGACACCGGC
INF_opaB_DW_R	TACCGAGCTCGAATTGTCGACCTCGCCCTCGGAGAGC
INF_opaC_UP_F	CCATGATTACGAATTATCACCATTCCGATGATGAAG
INF_opaC_UP_R	AGCTGGCGCTGCAGGGAATCATCGATGACGTGATG
INF_opaC_DW_F	ATCGATGACGTGATGCGCTC
INF_opaC_DW_R	TACCGAGCTCGAATTAAGGTGAGCGCGAACATCAAC
INF_opaD_UP_F	CCATGATTACGAATTAGCTCGTCGTTACGAACATGG
INF_opaD_UP_R	AGTCGGGTGCAGCGCCATGCACGGCCAACCGTGGG
INF_opaD_DW_F	GCGCTGCACCCGACTCGCGA
INF_opaD_DW_R	TACCGAGCTCGAATTGCCAATATCTCGTGGATGAAG
Complementary plasmids	
Co_opaAa_F	GAAGCTTCGTGGATCATGGCCGCGAGGACCGCATC
Co_opaAa_R	CAGGATATCTGGATCTGCCGGTGCTGGGGTCCTGG
Co_opaB_F	GAAGCTTCGTGGATCGACCTGCATTTCTGGACCTC
Co_opaB_R	CAGGATATCTGGATCAGGTGAGCGCGAACATCAAC
Co_opaC_F	GAAGCTTCGTGGATCTTCGCTGGTCGCCGTGATGG
Co_opaC_R	CAGGATATCTGGATCTTGCCGACGAATCGTCTGC
Co_opaD_F	GAAGCTTCGTGGATCAACAACGAAATTCGTCAGG
Co_opaD_R	CAGGATATCTGGATCGCTGTCATGGATATGTAC

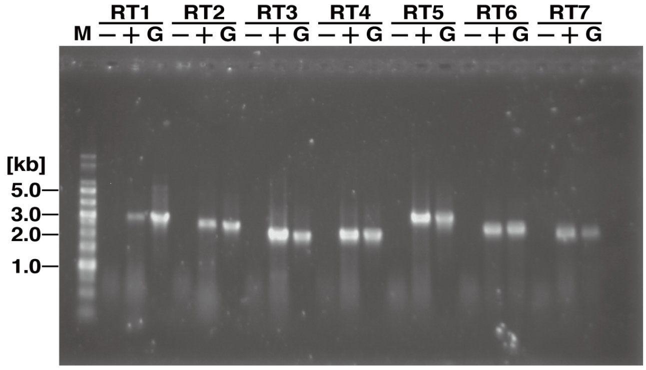

Fig. S4

Fig. S4. The results of agarose gel electrophoresis of RT-PCR products obtained with primers targeting RT1 (expected size 2,865 bp), RT2 (expected size 2,501 bp), RT3 (expected size 2,039 bp), RT4 (expected size 2,100 bp), RT5 (expected size 3,054 bp), RT6 (expected size 2,367 bp), and RT7 (expected size 2,124 bp) are shown. The amplified regions and the primer sequences are indicated in Fig. 1 (a) and Table S1, respectively. Lanes M, molecular size markers; lanes G, control PCR with the genomic DNA of strain PTH10; lanes + and -, RT-PCR with and without RT, respectively.
